# Five-year results from a prospective, single-arm European trial on decellularized allografts for aortic valve replacement—the ARISE Study and ARISE Registry Data

**DOI:** 10.1093/ejcts/ezae121

**Published:** 2024-03-26

**Authors:** Alexander Horke, Igor Tudorache, Günther Laufer, Martin Andreas, Jose Luis Pomar, Daniel Pereda, Eduard Quintana, Marta Sitges, Bart Meyns, Filip Rega, Mark Hazekamp, Robert Cesnjevar, Martin Oliver Schmiady, John Pepper, Ulrich Rosendahl, Artur Lichtenberg, Dmytro Stadnik, Ramadan Jashari, Dietmar Boethig, Dmitry Bobylev, Murat Avsar, Arjang Ruhparwar, Axel Haverich, Serghei Cebotari, Samir Sarikouch

**Affiliations:** Department for Cardiothoracic, Transplant, and Vascular Surgery, Hannover Medical School, Hannover, Germany; Clinic for Cardiac Surgery, University Heart Center Zurich, Zurich, Switzerland; Department of Cardiac Surgery, Medical University of Vienna, Vienna, Austria; Department of Cardiac Surgery, Medical University of Vienna, Vienna, Austria; Department of Cardiovascular Surgery, Hospital Clinico de Barcelona, Barcelona, Spain; Department of Cardiovascular Surgery, Hospital Clinico de Barcelona, Barcelona, Spain; Department of Cardiovascular Surgery, Hospital Clinico de Barcelona, Barcelona, Spain; Department of Cardiology, Hospital Clinico de Barcelona, Barcelona, Spain; Department of Cardiac Surgery, Katholieke Universiteit Leuven, Leuven, Belgium; Department of Cardiac Surgery, Katholieke Universiteit Leuven, Leuven, Belgium; Department of Cardiothoracic Surgery, Leiden University Medical Center, Leiden, Netherlands; Division of Congenital Cardiovascular Surgery, University Children’s Hospital, Zurich, Switzerland; Clinic for Cardiac Surgery, University Heart Center Zurich, Zurich, Switzerland; Division of Congenital Cardiovascular Surgery, University Children’s Hospital, Zurich, Switzerland; Department of Cardiovascular Surgery, Royal Brompton and Harefield NHS Foundation Trust, London, UK; Department of Cardiovascular Surgery, Royal Brompton and Harefield NHS Foundation Trust, London, UK; Department for Cardiac Surgery, University Hospital Düsseldorf, Medical Faculty of the Heinrich Heine University Düsseldorf, Düsseldorf, Germany; Department for Cardiac Surgery, University Hospital Düsseldorf, Medical Faculty of the Heinrich Heine University Düsseldorf, Düsseldorf, Germany; European Homograft Bank, Clinique Saint-Jean, Bruessels, Belgium; Department for Cardiothoracic, Transplant, and Vascular Surgery, Hannover Medical School, Hannover, Germany; Department for Cardiothoracic, Transplant, and Vascular Surgery, Hannover Medical School, Hannover, Germany; Department for Cardiothoracic, Transplant, and Vascular Surgery, Hannover Medical School, Hannover, Germany; Department for Cardiothoracic, Transplant, and Vascular Surgery, Hannover Medical School, Hannover, Germany; Department for Cardiothoracic, Transplant, and Vascular Surgery, Hannover Medical School, Hannover, Germany; Department of Cardiac Surgery, Institute for Cardiac Surgery and Interventional Cardiology, Luxembourg, Luxembourg; Department for Cardiothoracic, Transplant, and Vascular Surgery, Hannover Medical School, Hannover, Germany

**Keywords:** Aortic valve disease, Tissue engineering, Decellularization, Allografts

## Abstract

**OBJECTIVES:**

Decellularized aortic homografts (DAH) were introduced as a new option for aortic valve replacement for young patients.

**METHODS:**

A prospective, EU-funded, single-arm, multicentre study in 8 centres evaluating non-cryopreserved DAH for aortic valve replacement.

**RESULTS:**

A total of 144 patients (99 male) were prospectively enrolled in the ARISE Trial between October 2015 and October 2018 with a median age of 30.4 years [interquartile range (IQR) 15.9–55.1]; 45% had undergone previous cardiac operations, with 19% having 2 or more previous procedures. The mean implanted DAH diameter was 22.6 mm (standard deviation 2.4). The median operation duration was 312 min (IQR 234–417), the median cardiopulmonary bypass time was 154 min (IQR 118–212) and the median cross-clamp time 121 min (IQR 93–150). No postoperative bypass grafting or renal replacement therapy were required. Two early deaths occurred, 1 due to a LCA thrombus on day 3 and 1 due ventricular arrhythmia 5 h postoperation. There were 3 late deaths, 1 death due to endocarditis 4 months postoperatively and 2 unrelated deaths after 5 and 7 years due to cancer and Morbus Wegener resulting in a total mortality of 3.47%. After a median follow-up of 5.9 years [IQR 5.1–6.4, mean 5.5 years. (standard deviation 1.3) max. 7.6 years], the primary efficacy end-points peak gradient with median 11.0 mmHg (IQR 7.8–17.6) and regurgitation of median 0.5 (IQR 0–0.5) of grade 0–3 were excellent. At 5 years, freedom from death/reoperation/endocarditis/bleeding/thromboembolism were 97.9%/93.5%/96.4%/99.2%/99.3%, respectively.

**CONCLUSIONS:**

The 5-year results of the prospective multicentre ARISE trial continue to show DAH to be safe for aortic valve replacement with excellent haemodynamics.

**Figure:**
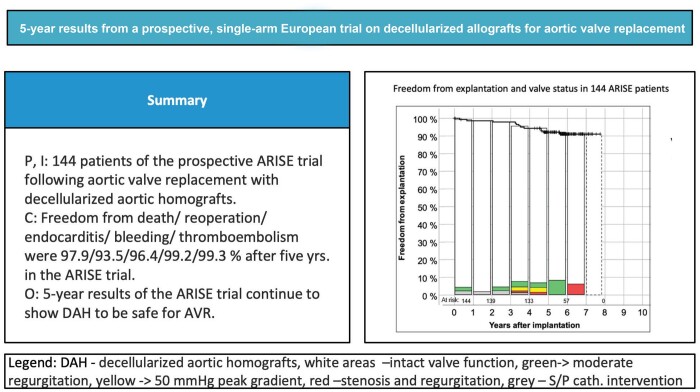

## INTRODUCTION

The Ross-autograft operation is frequently referenced in the literature as the gold standard for aortic valve replacement (AVR) in young patients, commonly defined as patients under 50 years of age [1–4]. In 2021, the Ross operation accounted for only 1.9% of all surgical AVR in Germany [5]. The results of the Ross operation therefore reflect an extremely selected patient cohort, which limits the transferability of the excellent study results to the individual patient.

The majority of young patients opt for alternative valve prostheses such as mechanical aortic valves. Mechanical valves provide excellent long-term durability and are the method of choice for young patients in most centres. A real challenge for these patients, if not a burden, are the inherent restrictions associated with the strict, life-long anticoagulation regime required to avoid mechanical valve thrombosis. In current meta-analyses, the risk for major adverse events while on this medication regime is estimated to be around 2% per patient-year, which is substantial given the young age of patients receiving mechanical prostheses [6]. Long-term survival following mechanical AVR was found to be significantly reduced in a recent meta-analysis, translating to a life expectancy of further 19 years for a 45-year-old patient compared to 34 years for the age-matched general population [6]. The possible reasons for the reduced life expectancy are manifold, for example patient–prosthesis mismatch leading to left ventricular failure and anticoagulation complications.

The reduced life expectancy may also reflect a negative patient selection, including patients who were not suitable Ross candidates due to cardiovascular issues and other co-morbidities, and also patients who had previously undergone several complicated cardiothoracic procedures. This is supported by the finding that no significant differences were observed between the 10- and 20-year mortality rates for Ross and non-Ross AVR in 25 young patients who had initially been considered as Ross candidates [7].

Biological heart valve prostheses, based on industrially processed xenogeneic material, are a further AVR option for young patients, which can lead to a better quality of life, as no anticoagulation is required. This is reflected in the high numbers of patients opting for this procedure: biological AVR prostheses account for 87% of all surgical AVR prostheses in Germany [5]. The drawbacks of these valves include higher reoperation rates, which may translate to higher long-term mortality rates as a result of repeated valve exchange procedures. However, in experienced hands, the mortality rates of a redo intra-annular AVR have been shown to be only slightly higher to those of the first AVR [8].

It should be noted that there is a significant difference in valve durability for a 20-year and a 50-year-old patient with a xenogenic biological heart valve prosthesis. In some very young patients, a rapid deterioration of valve function has even resulted in sudden cardiac death [9].

Cryopreserved human aortic valves are currently used predominantly for the treatment of extensive aortic root endocarditis due to their unique capacity to restore normal anatomy [10]. However, the durability of these conventional homografts in young patients is limited due to pronounced calcification, which can potentially lead to increased morbidity during redo operations [11].

Decellularized aortic homografts (DAH) may provide an additional AVR option for very young patients as they can potentially address the high failure rates of conventional allogenic and xenogeneic AVR prostheses [12, 13]. The near-normal haemodynamics in combination with the ability to repair a malfunctioning aortic root are especially important for patients with impaired myocardial function and patients with multiple previous aortic valve procedures [14]. Recent studies, however, suggested that an immunological, antibody-mediated response is present in almost all patients, which appears to be highly individual in terms of intensity [15, 16].

The aim of this study therefore is to (i) present the 5-year data from the 1st prospective, European-wide multicentre trial on DAH for AVR and (ii) to compare current DAH results from the ARISE Registry with contemporary data on the Ross procedure and other AVR options for young adults.

## MATERIALS AND METHODS

### Study setting

The ARISE Study received funding from the European Union’s HORIZON 2020 Programme under Grant Agreement No. 643597. The funding covered homograft procurement, homograft processing and data collection. Corlife oHG provided the decellularization service for the homografts and was the sponsor of the study according to Good Clinical Practice requirements.

The study was registered under ClinicalTrials.gov, NCT02527629 (‘Aortic Replacement Using Individualised Regenerative Allografts—ARISE the Surveillance’) and received the European Network of Centers for Pharmacoepidemiology and Pharmacovigilance (ENCePP^®^) seal as a Post Authorization Safety Study, EU PAS 10201. The study was also registered with the German Federal Institute for Vaccines and Biomedicines (www.pei.de) under Ref. Number NIS322. In addition, the ARISE Registry aims to follow all patients, who have received a decellularized aortic homograft for AVR before or outside the ARISE Study.

Approval was given by all local ethics committees prior to the start of the study, and informed consent was obtained appropriately from all participants or parents (MHH Nr. 2840-2015). The indication for AVR according to the current ESC/EACTS guidelines for valvular heart disease was the key inclusion criterion without age limits; patients with active endocarditis were excluded.

Patients were not included consecutively and patient selection was based on decision of the respective centre, the availability of an appropriate homograft and patient consent. Surgical procedures were performed under cardiopulmonary bypass according to locally established standard procedures. Postoperative anticoagulation with Warfarin was recommended for 2 months, followed by ASA at 100 mg per day to be continued as a permanent medication regime. Patients were followed annually by echocardiography at their respective centres or by their resident cardiologist. This applies also to patients within the ARISE Registry, where we aimed for annual echo data from resident paediatric or adult cardiologists.

The calculated sample size was 120 patients based on the average of 5.4% adverse clinical events per patient-year reported for mechanical valves and biological valves during follow-up, including sustained structural valve deterioration, nonstructural valve dysfunction, thromboembolism and bleeding, and endocarditis.

The primary end-points were (i) periprocedural complications (all-cause mortality, major stroke, life-threatening or disabling bleeding, acute kidney injury requiring renal replacement therapy, myocardial infarction, major vascular complications), (ii) heart valve dysfunction (aortic valve area ≤1.2 cm^2^ and mean aortic valve gradient ≥20 mmHg or peak velocity ≥3 m/s, or moderate or severe prosthetic valve aortic regurgitation) and (iii) repeat procedure for valve-related dysfunction (surgical or interventional therapy).

### Homograft procurement and processing

Homografts were procured in line with the current European Directive 2004/23, as amended, via 4 different tissue banks (European Homograft Bank, Brussels, Dr R. Jashari; German Society for Tissue Transplantation—DGFG, Hannover, M. Börgel; EuroTissue Bank, Rotterdam, A. van den Bogaerdt; Banc de Sang I Teixits, Barcelona, Dr E. Trias) and shipped to Hannover for processing at Corlife (www.corlife.eu).

DAH was authorized by the German competent authority as ‘Cell-free aortic heart valve, Arise AV’, # PEI.G.11766.0.1. The processing of each homograft comprises ∼30 different steps using a detergent-based, non-cryopreservation approach as described previously [17]. Microbiological assessment was performed as part of the incoming inspection, both during and after processing with a final 14-day quarantine. Each homograft was assessed histologically following processing, and the residual dsDNA content was measured before and after processing prior to final release. Reference samples of all homografts were stored in accordance with German law for at least 1 year. Processed homografts were stored at 4°C and were acceptable for implantation until 180 days after tissue procurement.

### Statistics

Summaries of numeric data are given as means and standard deviation. The proportion of explanted and dysfunctional grafts over time was calculated and a peak echocardiographic gradient of ≥50 mmHg and regurgitation greater than or equal to moderate was defined as dysfunctional.

Time-related events, such as freedom from explantation and degeneration, were evaluated according to Kaplan–Meier. We did not assess the competing risks of death and heart valve dysfunction as mortality was very low.

We calculated perioperative and annual adverse events such as death, reoperation or reintervention, valve degeneration, thrombotic and bleeding events, and endocarditis for all DAH implanted to date from the ARISE Registry, which has a 90% follow-up of all patients. These results were compared with those from recent large-scale meta-analyses for bioprostheses (*n* = 2686), mechanical valves (*n* = 5728) and the Ross procedure in young adults (*n* = 6892), provided by the group of Johanna Takkenberg [2, 6, 18].

A long-term estimation was performed by adding the reported [2, 6, 18] early adverse event rates and the indicated linearized annual adverse event rates beyond the early postoperative time. For DAHs, we added the observed early adverse events and late annual events in the same categories as provided within the meta-analyses for the other aortic valve substitutes. Group- and pairwise confidence interval calculations and comparisons based on estimated patient numbers and observed early and yearly complication rates were performed and are explained and illustrated in detail within Supplementary Material, Appendix A.

We refrained from claiming statistical significance, as long-term DAH follow-up still is limited and as we compared prospectively collected, multicentre DAH data with meta-analyses summarizing predominantly retrospective and single-centre studies.

SPSS 28 (IBM Corporation, Somer, NY), R (R version 4.2.2; 31 October 2022, The R Foundation for Statistical Computing) and RStudio (RStudio Team 2020, PBC, Boston, MA) and EZR Version 1.61 were used for the analyses and the graphical illustrations of the results.

## RESULTS

### Perioperative outcome

One-hundred forty-four patients (99 male, 69%) were prospectively enrolled in the ARISE Trial between October 2015 and October 2018 with a mean age of 33.6 years [standard deviation (SD) 20.8, median 30.4 years, interquartile range (IQR) 15.9–55.1]. Forty (28%) were paediatric patients and 65 (45%) of the patients had undergone previous cardiac operations. Twenty-seven (19%) had undergone 2 or more previous surgical procedures. In 24 patients (16.7%), a prosthetic aortic valve was replaced with DAH. The mean implanted DAH diameter was 22.6 mm (SD 2.4). The median operation duration was 312 min (IQR 234–417), the median cardiopulmonary bypass time was 154 min (IQR 118–212) and the median cross-clamp time was 121 min (IQR 93–150). No postoperative bypass grafting or renal replacement therapy was required. DAH were implanted in full-root technique all but one, which was implanted in subcoronary position.

Two early deaths occurred; 1 due to a LCA thrombus on day 3 and 1 due to ventricular arrhythmia 5 h postoperation. There were 3 late deaths. One death occurred due to endocarditis 4 months postoperatively and 2 unrelated deaths occurred after 5 years due to cancer and after 7 years due to Wegener’s granulomatosis with polyangiitis, resulting in a total mortality of 3.47%.

One pacemaker implantation was necessary for postoperative atrioventricular block and 1 DAH was repaired due to early regurgitation after 6 weeks.

### Follow-up results within the prospective ARISE Study cohort

The follow-up was 100% complete within the prospective ARISE Study cohort. After a median follow-up of 5.9 years [IQR 5.1–6.4, mean 5.5 years (SD 1.3), max. 7.6 years], the primary haemodynamic end-points peak gradient with a median of 11.0 mmHg (IQR 7.8–17.6) and regurgitation of a median 0.5 (IQR 0–0.5) of grade 0–3 were excellent (0: no regurgitation, 0.5: trace, 1.0: mild, 1.5: mild to moderate, 2.0: moderate, 2.5: moderate to severe and 3.0: severe).

**Figure 1:**
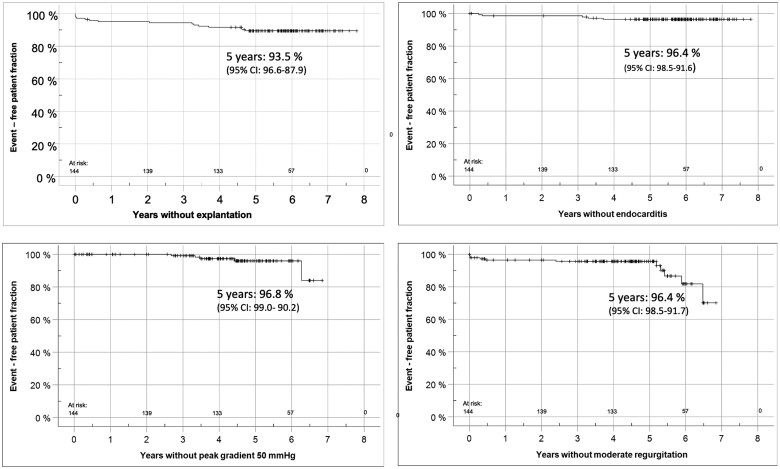
This figure shows freedom from homograft explantation, endocarditis and valve degeneration for the ARISE cohort as Kaplan–Meier curves.

At 5 years, the rates of freedom from death/reoperation/endocarditis/bleeding/thromboembolism were 97.9%/93.5%/96.4%/99.2%/99.3%, respectively. Figure 1 shows the rates of freedom from homograft explantation, endocarditis and valve degeneration as Kaplan–Meier curves.

**Figure 2:**
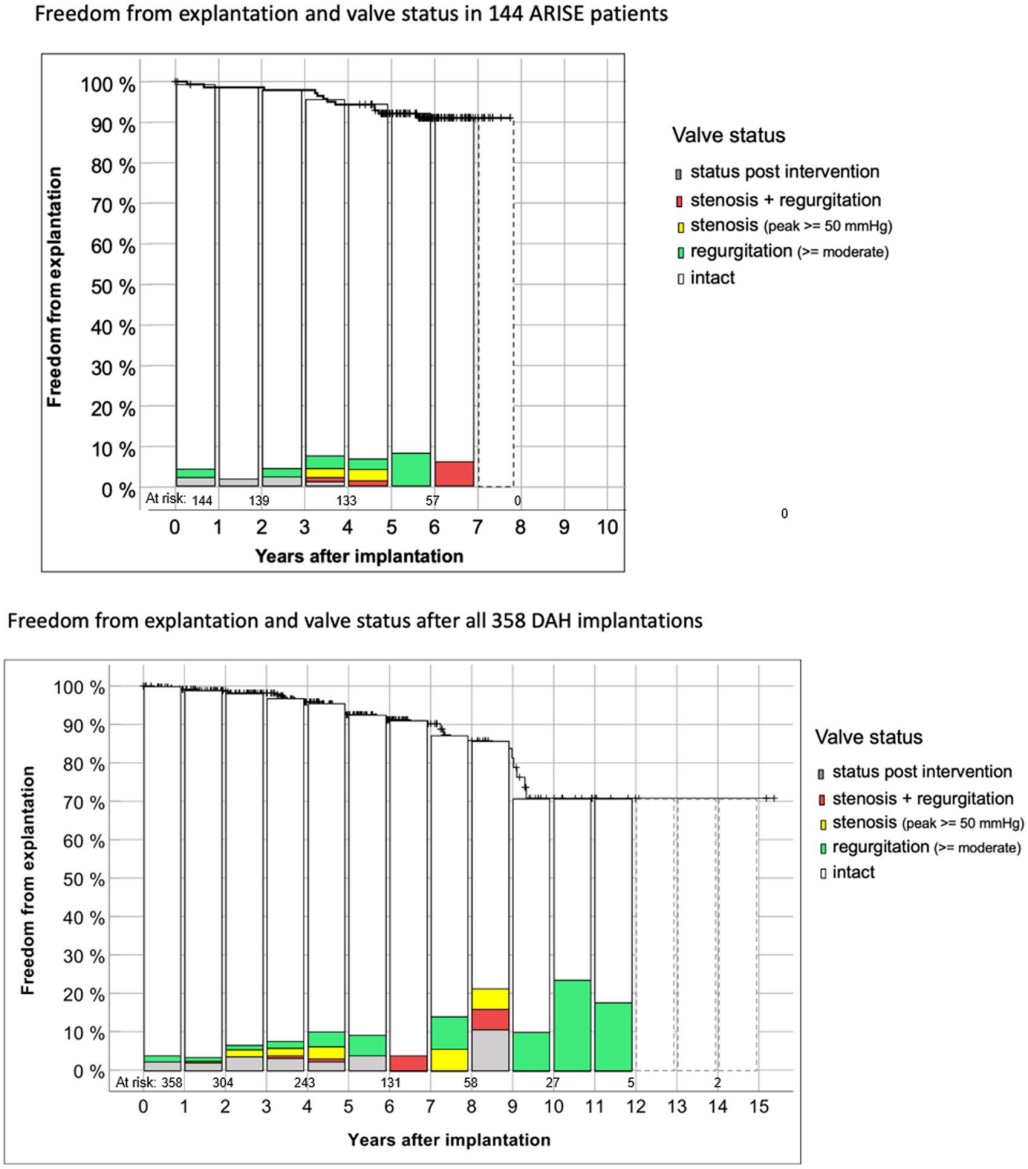
Details on freedom from explantation according to Kaplan–Meier and the functional status in the 144 ARISE patients and in all 358 implanted DAH to date with up to 15 years of follow-up. The dashed boxes in the ARISE patients indicate patients with missing echo data for 2023. The dashed boxes for the whole DAH group indicate the years in which follow-ups in Moldovia could only be performed via telephone due to the COVID19 pandemic and the war in the Ukraine. DAH: decellularized aortic homografts.

Table 1 displays patient characteristics for the ARISE Study and the total ARISE Registry cohorts. Figure 2 shows freedom from explantation according to Kaplan–Meier and the functional status in the 144 ARISE patients and in all 358 DAHs implanted to date with up to 15 years of follow-up. The dashed boxes in the ARISE patients indicate patients with outstanding echo data for 2023. The dashed boxes for the whole DAH group indicate the years in which follow-ups in Moldovia could only be performed via telephone due to the COVID19 pandemic and the war in the Ukraine.

**Table 1:** Patient characteristics for the ARISE Study and the total ARISE Registry cohort

	ARISE study cohort, *n* = 144	All DAH, *n* = 358
Implantation period	2015–2018	2008–2023
Age at implantation (years)	30.4 [15.9–55.1]	23.9 [12.5–44.8]
Follow-up, mean (years)	5.9 [5.1–6.4]	5.0 [3.1–6.5]
Total follow-up (years)	789	1748
Sex (male)	99 (69%)	250 (70%)
Number of previous operations		
0	79	200
1	38	90
2	19	46
>2	8	22
Type of previous procedures		
1 × aortic valve replacement	19	49
2 × aortic valve replacement	5	11
3 × aortic valve replacement	23	2
Catheter-based intervention		92
Aortic valve repair	6	40
Mean allograft diameter (mm)	22.6 (2.4)	23 [21–25]
10–18	9	28
19–22	56	127
23–29	79	203
Implantation time (min)		
Total operation	312 [234–417]	303 [244–400]
Cardiopulmonary bypass	154 [118–212]	168 [132–220]
Cross clamp	121 [93–150]	120 [97–146]
Latest echocardiography		
Aortic annulus (mm)	22.6 (2.6)	22.1 (3.8)
Aortic annulus, z-score	–0.19 (1.3)	0.18 (1.47)
Effective orifice area (cm^2^)	3.1 (0.9)	2.95 (0.83)
Peak gradient (mmHg)	11.0 [7.8–17.6]	11.6 [7.8–19.4]
Regurgitation (grade 0–3)	0.5 [0-0.5]	0.5 [0–1]
LV ejection fraction (%)	65.5 [61.3–67.0]	64.0 [59.5–68.0]

Mean and standard deviation are shown in round brackets for normally distributed factors, median and interquartile range (IQR) are shown in square brackets for factors with no normal distribution.

DAH: decellularized aortic homografts; LV: left ventricle.

### Comparison with published Ross-autograft procedure cohorts

Table 2 lists freedom from diverse adverse outcomes within the ARISE Study (*n* = 144) and the ARISE Registry (*n* = 358) compared with the Ross cohort published by David *et al.* (*n* = 212) [19] and a large recent Ross review provided by Takkenberg *et al.* [2] summarizing published results from 6892 adult patients.

**Table 2:** Freedom from diverse adverse outcomes for decellularized aortic homografts (DAH) within the ARISE Study and the ARISE Registry, both including children, compared with the Ross cohort published by David *et al*. [19] and a large Ross review published by Takkenberg *et al*. [2]

Freedom from (%)	Cohort	At 5 years (mean ± SD in %)	At 10 years (mean ± SD in %)
Death	ARISE_(n=144)_	97.9 (1.0)	n.a.
	All DAH_(__*n*__= 358)_	98.3 (0.7)	95.7 (1.7)
	Ross_(__*n*__= 212)_ [19]	98.6	97.6
	Ross_(__*n*__= 8523)_ [2]	96.2	93.9
Endocarditis	ARISE	96.4 (1.6)	n.a.
	All DAH	97.8 (0.9)	92.6 (3.1)
	Ross	100	98.6
	Ross	n.a.	n.a.
Explantation/Re-operation	ARISE	93.5 (2.1)	n.a.
	All DAH	92.4 (1.7)	69.5 (6.5)^a^
	Ross	97.1	94.2
	Ross_(__*n*__= 6653)_ [2]	92.1	80.9
Major bleeding	ARISE	98.6 (1.0)	n.a.
	All DAH	99.4 (0.4)	98.8 ± 0.9
	Ross	n.a.	n.a.
	Ross	n.a.	n.a.
Thrombotic event/stroke	ARISE	99.3 (0.1)	n.a.
	All DAH	99.7 (0.3)	94.4 (5.1)
	Ross	100	100
	Ross	n.a.	n.a.

a Freedom from explantation in all adult patients within the ARISE Registry was 72.8% (SD 10.0).

SD: standard deviation; n.a.: not available.

DAH patients were younger with a median of 30.4 years (IQR 15.6–55.1) versus median 34 years (IQR 28–41) [19] and 41.9 years (SD 11.4) [2] with paediatric patients comprising 40% (*n* = 143) of the ARISE Registry cohort.

### Expected adverse event calculation for contemporary aortic valve replacement options for young adults

Perioperative and annual adverse events such as death, any reoperation or reintervention, valve degeneration, thrombotic and bleeding events and endocarditis were compared for conventional aortic bioprostheses, mechanical valves, the Ross procedure and DAH to provide an overview of expected adverse events per patient.

To avoid confounding effects resulting from patient age, we limited this analysis to data from adult DAH patients, and 215 adult DAH patients with a median of 39.2 years (IQR 27.6–53.1) were followed within the ARISE Registry.

**Figure 3:**
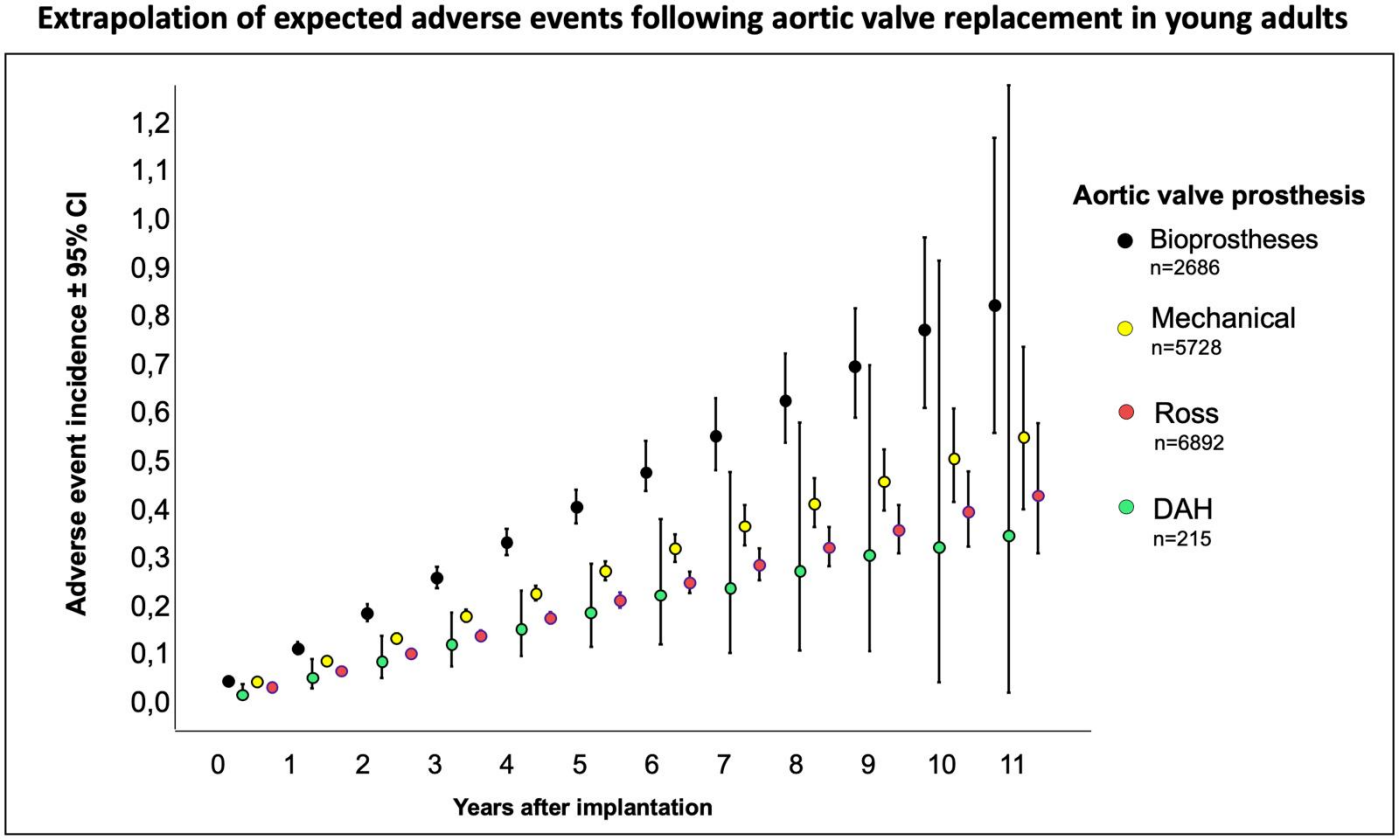
ARISE Registry Data of all 215 DAH implanted in young adults compared with recently published meta-analysis data from several AVR options in young adult patients. Perioperative and linearized annual adverse events such as death, reoperation or reintervention, valve degeneration, thrombotic and bleeding events, and endocarditis are summarized to provide an estimate of adverse events in the long-term. In order to estimate the statistical significance of these performance comparisons, we assumed a decrease in the numbers of patients at risk proportional to the decrease in our DAH population. Multiplying expected adverse event rates and estimated patient numbers resulted in event incidence rates. Poisson distribution based 95% confidence intervals were calculated for each year and valve group. Ross results include right ventricular procedures. Other data were taken from recent large-scale meta-analyses from Takkenberg *et al*. [5, 16, 2].

Table 3 and Fig. 3 show DAH results in young adults in comparison to the conventional alternatives of mechanical valves, the Ross procedure and standard bioprostheses. Other data were taken from recent large-scale meta-analyses from Takkenberg *et al.* [2, 5, 16]. Ross results include right ventricular procedures. Supplementary Material, Appendix A provides a detailed description of this comparison.

**Table 3:** Observed perioperative mortality and annual adverse events for all 215 DAH in young adults in comparison with reported results from Ross procedures, mechanical AVR and standard allograft implantation in young adults

	DAH	Ross^a^	Mechanical^b^	Bioprosthesis^c^
Early death %	0.93	2.01	3.15	3.30
Late mortality %/y	0.29	0.59	1.55	2.39
Any reoperation and intervention %/y	0.95	1.20	0.51	1.82
Valve degeneration %/y	1.52	1.3	0.39	1.83
Thrombosis/bleeding %/y	0.19	0.30	1.89	0.82
Endocarditis %/y	0.48	0.27	0.41	0.48

Ross results include right ventricular procedures.

a Data taken from Etnel *et al*. *Circ Cardiovasc Qual Outcomes* 2019;12:e005481.

b Data taken from Korteland *et al*. *Eur Heart J*. 2017; 38:3370–7.

c Data taken from Etnel *et al*. *Circ Cardiovasc Qual Outcomes* 2018;11:e004748.

## DISCUSSION

The five-year data from the only prospective trial on DAH for AVR to date show excellent results in the chosen European multicentre setting. Haemodynamic results and freedom from adverse events such as death, endocarditis or valve degeneration at 5 years compared well with published results from experienced Ross groups, e.g. from the Toronto General Hospital by David *et al.* [19]. It is important to note that these results were matched despite the ARISE cohort having a younger patient age, twice as many previous cardiac procedures and 5 times the number of previous AVR procedures. While advances in surgical interventions must be taken into account here, as the procedures for the Toronto patients were performed 2 decades ago, the results nevertheless are a clear indication of the safety and potential of DAH.

A comparison of adverse events for adult DAH patients from the ARISE Registry with published results from recent large-scale meta-analyses provided by Takkenberg *et al.* [2, 6, 18] showed comparable results between DAH and Ross procedures and better results than those obtained with mechanical AVR and bioprostheses. Freedom from death within the ARISE Registry at 10 years was 95.7% and thereby better than the reported results for decellularized and cryopreserved aortic homografts (76% and 57%) provided by CryoLife, Inc, Kennesaw, GA [20] and similar to the Ross results (97%) within the only randomized clinical trial comparing the Ross operation and homografts [4].

While these overall results are very good, the functional long-term data at 10 years also show a steady increase in patients presenting with a malfunction of the implanted DAH. As the proportion of degenerative DAH is clearly higher in paediatric patients [12], we hypothesize that, despite thorough decellularization, DAH remain immunogenic, thereby leading to thickening of the cusps and wall calcification. To date, there is no information available about which exact antigene induces the immune response in DAH. We have shown highly individual humoral responses towards decellularized tissue *in vitro* and have initiated prospective serial assessment *in vivo* following implantation of decellularized aortic and pulmonary homografts [15, 16]. Current hypotheses point towards so-called matrikines, specific peptides of the extracellular matrix that may result from the decellularization process as a target for the host immune system [21]. New cytokine and chemokine analysis of explanted DAH (in preparation) indicate irregular activation of fibrotic repair mechanisms and no classic T-cell-mediated immune response, which supports our histological results, in which no T-cell invasion was observed [22]. Delineating the patho-mechanism is paramount for the development of advanced decellularization protocols and for camouflage by new silencing techniques. Understanding the immune response towards decellularized allografts would also help research towards decellularized xenografts, which has reached large animal experiment level.

The best option for AVR remains a challenging choice for the individual patient. The present analysis adds important information as a basis for this decision as it shows good results for DAH in patients who have undergone a considerable number of previous cardiac procedures. From a surgical perspective, patient anatomy is the most important factor in the choice of the respective aortic prosthesis. Therefore, even propensity score-matched retrospective comparisons of the proposed gold standard Ross procedure to bioprostheses and mechanical AVR are unhelpful if they do not consider patient specifics, such as annular dimensions and/or previous surgical procedures [23]. The only truly valid comparison in our opinion would be a prospective randomized trial, comparing mechanical AVR, Ross and DAH. We recently submitted a proposal for this type of trial to the European Commission, which was unfortunately not selected for funding.

Current DAH results fall short of initial expectations, as full integration has thus far not been reliably achieved, which, in turn, entails foreseeable re-operations for patients. However, we see DAH as a valid option for patients who are not good candidates for a Ross procedure due to their anatomy or previous surgical procedures. The question of whether DAH is better than an intra-annular procedure with a mechanical valve remains open. The main disadvantage of mechanical AVR is the need for permanent anticoagulation, but the surgical procedure is much simpler as no coronary re-implantation is required, as is the case for DAH. Reported early and late mortality for mechanical AVR, however, is higher than the rates we have observed for DAH so far and long-term adverse event estimations, including late mortality, show favourable results for DAH.

### Limitations

The duration of follow-up data currently available for DAH patients and the comparably small overall number of patients treated so far are clear limitations of the present study, and definitive conclusions will require a longer follow-up period.

Further limitations include the one-armed study design and the restrictions inherent in the comparison of prospectively collected data from a controlled trial with retrospectively conducted single-centre analyses and meta-analyses based on such reports and potential currently unknown confounders.

## CONCLUSION

At 5-years, follow-up of the prospective multicentre ARISE Trial shows excellent haemodynamic function of DAH.

DAH results in young adults compared well with contemporary adult Ross cohorts, but longer follow-up is crucial for definitive conclusions. Ten-year follow-up data within the ARISE Registry, which includes 40% children, suggest residual immunogenicity leading to reoperation.

## Supplementary Material

ezae121_Supplementary_Data

## Data Availability

The data underlying this article will be shared on reasonable request to the corresponding author. **Alexander Horke:** Data curation; Formal analysis; Investigation; Resources; Writing—original draft. **Igor Tudorache:** Conceptualization; Data curation; Investigation; Writing—review and editing. **Günther Laufer:** Data curation; Investigation; Resources; Writing—review and editing. **Martin Andreas:** Data curation; Investigation; Writing—review and editing. **Jose Luis Pomar:** Data curation; Investigation; Project administration; Writing—review and editing. **Daniel Pereda:** Data curation; Investigation; Writing—review and editing. **Eduard Quintana:** Data curation; Investigation; Writing—review and editing. **Marta Sitges:** Data curation; Investigation; Writing—review and editing. **Bart Meyns:** Data curation; Investigation; Resources; Writing—review and editing. **Filip Rega:** Data curation; Investigation; Writing—review and editing. **Mark Hazekamp:** Data curation; Investigation; Resources; Writing—review and editing. **Robert Cesnjevar:** Data curation; Investigation; Resources; Writing—review and editing. **Martin Oliver Schmiady:** Data curation; Investigation; Writing—review and editing. **John Pepper:** Data curation; Investigation; Resources; Writing—review and editing. **Ulrich Rosendahl:** Data curation; Investigation; Writing—review and editing. **Artur Lichtenberg:** Data curation; Investigation; Resources; Writing—review and editing. **Dmytro Stadnik:** Data curation; Investigation; Writing—review and editing. **Ramadan Jashari:** Conceptualization; Investigation; Resources; Writing—review and editing. **Dietmar Boethig:** Data curation; Formal analysis; Software; Visualization; Writing—review and editing. **Dmitry Bobylev:** Data curation; Investigation; Writing—review and editing. **Murat Avsar:** Data curation; Investigation; Writing—review and editing. **Arjang Ruhparwar:** Investigation; Resources; Writing—review and editing. **Axel Haverich:** Conceptualization; Data curation; Funding acquisition; Methodology; Project administration; Writing—review and editing. **Serghei Cebotari:** Conceptualization; Data curation; Investigation; Writing—review and editing. **Samir Sarikouch:** Conceptualization; Data curation; Formal analysis; Funding acquisition; Investigation; Methodology; Project administration; Writing—original draft. European Journal of Cardio-Thoracic Surgery thanks Jan Vojacek, William T Brinkman, Paul Stelzer and Gebrine El Khoury for their contribution to the peer review process of this article.

## Notes

Presented at the 2023 EACTS, Vienna, Austria.

## ABBREVIATIONS

AVR: Aortic valve replacement
IQR: Interquartile range
SD: Standard deviation
