# Supplementary figures and images for: Five-year results from a prospective, single-arm European trial on decellularized allografts for aortic valve replacement—the ARISE Study and ARISE Registry Data
Source: Eur J Cardiothorac Surg. 2024 Mar 26;65(4):ezae121. doi: 10.1093/ejcts/ezae121 (PMC11009017; doi:10.1093/ejcts/ezae121)

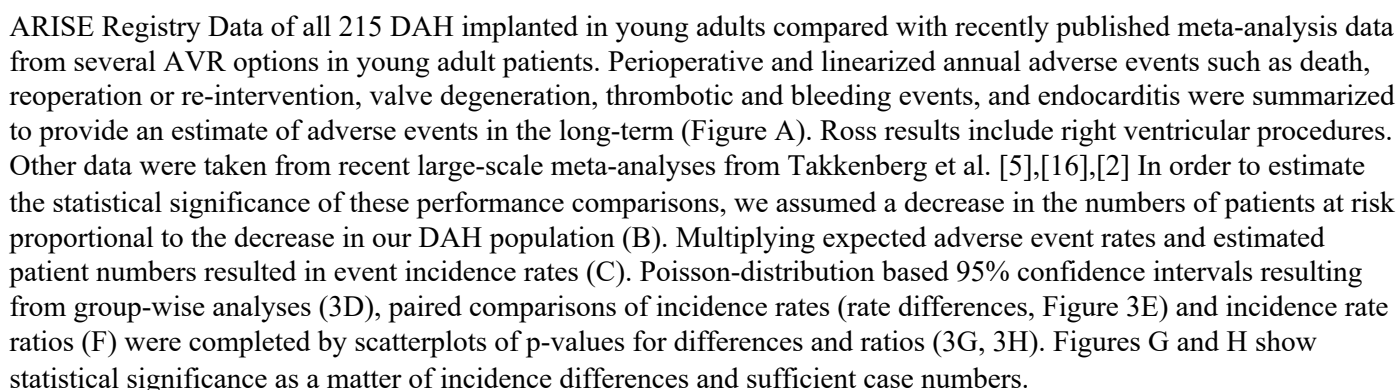

Supplement: ezae121_Supplementary_Data [file ezae121_supplementary_data.pdf]
